# Extra Corporeal Membrane Oxygenation in the Treatment of Human Immunodeficiency Virus-Related *P. jirovecii* Pneumonia

**DOI:** 10.3390/idr13040092

**Published:** 2021-12-02

**Authors:** Sara Lacerda Pereira, Elsa Branco, Ana Sofia Faustino, Paulo Figueiredo, António Sarmento, Lurdes Santos

**Affiliations:** Centro Hospitalar e Universitário de São João, Alameda Professor Hernâni Monteiro, 4200-319 Porto, Portugal; elsa.alves.branco@gmail.com (E.B.); asfaustino@gmail.com (A.S.F.); pfigueiredodias@hotmail.com (P.F.); antonio.sarmento@chsj.min-saude.pt (A.S.); maria.lurdes.uci@gmail.com (L.S.)

**Keywords:** extra corporeal membrane oxygenation, *Pneumocystis jirovecii* pneumonia, human immunodeficiency virus, respiratory insufficiency, critical care, ventilator induced lung injury

## Abstract

Despite the undeniable complexity one may encounter while managing critically ill patients with human immunodeficiency virus infection (HIV), intensive care unit-related mortality has declined in recent years, not only because of more efficacious antiretroviral therapy (ART) but also due to the advances in critical support. However, the use of extracorporeal membrane oxygenation (ECMO) in these patients remains controversial. We report four cases of HIV-infected patients with *Pneumocystis jirovecii* pneumonia (PJP) and acute respiratory distress syndrome (ARDS) treated with ECMO support and discuss its indications and possible role in the prevention of barotrauma and ventilator- induced lung injury (VILI). The eventually favorable clinical course of the patients that we present suggests that although immune status is an important aspect in the decision to initiate ECMO support, this technology can provide real benefit in some patients with severe HIV-related refractory ARDS.

## 1. Introduction

During the human immunodeficiency virus (HIV) pandemic in the early 1980s, critical illness in HIV-infected patients was associated with poor prognosis with high morbidity and mortality [1]. Available therapies for HIV were limited. The main cause of Intensive Care Unit (ICU) admission in this population was, and still is, respiratory failure due to *Pneumocystis jirovecii* pneumonia (PJP), which was associated with remarkably high mortality (70 to 90%). Consequently, ICU admission of HIV-positive patients at that time was discouraged and considered worthless [2,3,4].

With the widespread availability of antiretroviral therapy (ART), the overall survival of these patients has progressively improved, including those admitted to the ICU [3,5,6,7]. Consequently, the criteria for intensive care treatment for acutely ill HIV-infected patients have changed over recent years, although not uniformly [1]. Sepsis and severe respiratory failure (SRF) are still the most common reasons for ICU admission in patients with HIV, mainly as a consequence of bacterial community-acquired pneumonia (CAP) and PJP [5,6,7,8,9].

Although opportunistic infections besides PJP and other acquired immunodeficiency syndrome (AIDS)-related illnesses are responsible for a smaller proportion of ICU admissions, they are still encountered, especially in late presenters with de novo HIV diagnosis. Moreover, advances in general ICU care may also explain the survival gains in critically ill HIV-positive individuals, namely, improved knowledge of invasive mechanical ventilation (IMV), the use of noninvasive ventilation for PJP, aggressive management of sepsis and treatment of organ dysfunctions as well as the early admission of patients to the ICU. Nevertheless, the need for invasive mechanical ventilation in HIV-infected patients remains an indicator of poor outcome and increased mortality [1]. Extra corporeal membrane oxygenation (ECMO) is a form of cardiac and/or pulmonary life-support by which blood is drained from the native vascular system, circulated through a pump that moves blood forward, passed through an external gas-exchanging membrane responsible for oxygenation and carbon dioxide removal and is then reinfused into the circulation. It can provide both respiratory and hemodynamic support depending on the location of the drainage and return. Its utilization became more widespread after the H1N1 influenza pandemic [10]. In patients with refractory life-threatening hypoxemia or hypercapnia during IMV, ECMO can be lifesaving [10,11,12].

Several parameters are used to calculate the probability of death before ECMO and to help in the decision regarding its initiation, such as ventilator settings, the cause of ARDS, the age and immune status of the patient, gas exchange at baseline, use of pre-ECMO rescue therapies, organ disfunction scores (like the APACHE, SOFA and SAPS II scores) and the Murray score [10,11].

In the critical care setting it is frequently difficult to predict those patients who will benefit from extracorporeal life-support interventions when the chances of a successful outcome are unpredictable. Traditionally, immunocompromised status has been considered a relative contraindication to the initiation of ECMO, because ARDS treatment in immunocompromised patients is particularly challenging due to poor organ recovery, infectious complications and hemorrhagic and thrombotic issues, among others [5,11]. An additional important reason to use ECMO in patients with PJP is the remarkably high incidence of barotrauma in that population, which is a life- threatening complication with the use of positive pressure ventilation. During ECMO, the protective ventilation strategy can be employed without worrying about hypoxemia and carbon dioxide retention [5,13].

In this review, we present our experience with ECMO support in HIV-infected patients with AIDS and discuss its indications and possible role in the prevention of barotrauma in cases of PJP and ARDS.

## 2. Case Reports

### 2.1. Case 1

**Figure 1:**
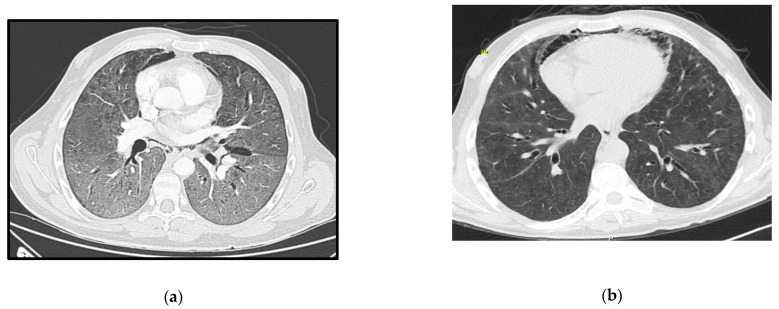
Case 1 thoracic CT-scan at diagnosis (**a**) and follow-up (**b**).

A 29-year-old male, HIV-positive since 2015, severely immunosuppressed that was lost to follow-up before starting ART. He presented in March 2019 at the emergency room (ER) with a one-day history of fever, shortness of breath and cough without providing information about his HIV status. Initial assessment showed polypnea of 30 cycles per minute (cpm), hypoxia, fever (39 °C), elevated C-Reactive Protein (CRP) and bilateral middle and lower zone air space opacities on chest X-ray. He was admitted to the ward and started empirical treatment for community acquired pneumonia (CAP). Two days later, he was transferred to the ICU with aggravated tachypnea (50 cpm), severe hypoxemia (paO_2_ 49 mmHg) despite oxygen supplementation and pneumomediastinum, bilateral pneumothorax and diffuse ground-glass opacities on thoracic-CT scan (Figure 1a). The CD4+ lymphocyte count was 6/mm^3^ and the HIV-viral load was 18,200 copies/mL. All other microbiologic tests were negative. Treatment was then switched empirically to trimethoprim-sulfamethoxazole (TMP-SMX) 15 mg/kg of TMP each day in 3 takes plus corticosteroids for a presumed diagnosis of PJP. Later the diagnosis was confirmed by positive immunofluorescence as *Pneumocystis jirovecii* (*P. jirovecii*) in bronchoalveolar fluid (BAL).

Due to refractory hypoxemia and given the high probability of barotrauma, the patient was started on venovenous-ECMO(VV-ECMO) without prior tracheal intubation. He later needed intubation due to poor bronchial clearance of secretions and completed a 14 days-period of protective IMV in an attempt to reduce extra corporeal support. He completed 21 days of therapy with TMP-SMX plus corticosteroids according to recommended PJP treatment dosage (prednisolone 40 mg two times day for 5 days, then 40 mg each day for 5 days and after that 20 mg each day for 11 days). ART was started 15 days after the ICU admission, with a significant reduction in the viral load one month later (151 copies/mL). ECMO and protective IMV were maintained for 40 days, followed by 19 days of weaning off. The pneumomediastinum and bilateral pneumothorax were managed conservatively, and a new CT-scan performed 50 days later showed great improvement (Figure 1b). He was transferred to the ward after 69 days of ICU stay showing signs of significant myopathy. Three months after discharge, he was revaluated at outpatient care as fully recovered and with CD4+ lymphocyte count improvement (49/mm^3^).

### 2.2. Case 2

**Figure 2:**
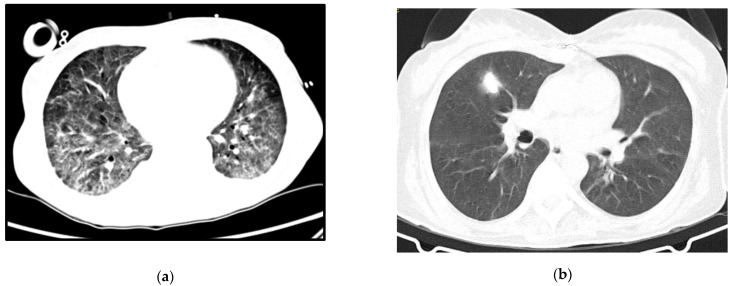
Case 2 thoracic CT-scan at diagnosis (**a**) and follow-up (**b**).

A 64-year-old woman with a history of hypertension, dyslipidemia and chronic pulmonary disease presented at the ER with fever, shortness of breath and a worsening cough despite a previous complete course of antibiotics for presumed CAP. She was hypoxic, with isolated elevation of CRP and diffuse ground-glass opacities on thoracic CT-scan (Figure 2a). Her status deteriorated despite antibiotics and oxygen supplementation in the Intermediate Care Unit, so she was transferred to the ICU and intubated. Three days after IMV and prone positioning, she was connected to VV-ECMO due to refractory respiratory acidemia. Anti-HIV testing was positive. Immune and viral study revealed severe immunosuppression (9 CD4+/mm^3^) and high serum viral load (4.050.000 copies/mL) and TMP-SMX plus corticosteroids were started for presumed PJP, at the recommended PJP treatment dosage. Diagnosis was confirmed by positive immunofluorescence for *P. jirovecii* in BAL.

ECMO was discontinued after 10 days. During the weaning off invasive ventilation, there was recrudescence of ARDS with increased ventilatory parameters and need for prone positioning. Nosocomial infection was considered, broad spectrum antibiotics were started and bronchofibroscopy repeated, with persistently positive immunofluorescence for *P. jirovecii* and a positive polymerase chain reaction (PCR) for cytomegalovirus in BAL. She completed a total of 33 days of treatment with TMP-SMX and 21 days of ganciclovir with respiratory improvement and started ART. She was extubated after 83 days and was transferred to the ward after three months of ICU stay for muscular rehabilitation, without other dysfunctions.

Follow-up imaging can be seen in Figure 2b. She was transferred to a rehabilitation unit with a residual need of oxygen support (2 L per minute), from which she recovered after some months of pulmonary rehabilitation.

### 2.3. Case 3

**Figure 3:**
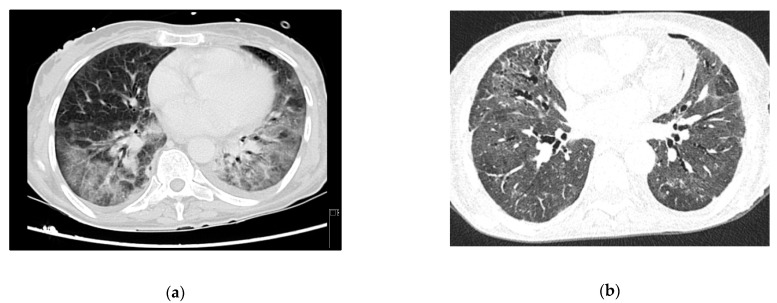
Case 3 thoracic CT-scan at diagnosis (**a**) and follow-up (**b**).

A 53-year-old woman, with no relevant medical history so far, was brought to the ER due to a two-month history of progressive psychomotor slowness and confusion, which had worsened in the week before. At physical examination, she was agitated and febrile. Head CT scan showed some intra-axial lesions in the left frontal and temporal lobes. The cerebral spinal fluid (CSF) had mild pleocytosis and moderately elevated proteins. The serology for HIV was positive, and the nucleic acid test of the CSF was positive for toxoplasma gondii. She was admitted in the ICU with a de novo diagnosis of HIV infection, with severe immunosuppression (CD4+ count 28 cells/mm^3^), clinically manifested as cerebral toxoplasmosis. On day 3, she began coughing, with respiratory hypoxemic insufficiency and bilateral diffuse glass opacities on chest-CT scan (Figure 3a). The presumptive diagnosis of PJP was posteriorly confirmed with both direct dye-examination and PCR positive for *P. jirovecii* in BAL. She was treated with TMP-SMX for both PJP and cerebral toxoplasmosis. Following one week of appropriate medical treatment, the patient had a favorable response, and was discharged to the ward for further care.

At the end of the month, she was readmitted to the ICU because of respiratory failure and elevated lactate. Respiratory secretions and gastric aspirate were both negative for tuberculosis. Other microbiology tests (including blood serologies for other common opportunistic agents) were also negative. She repeated chest-CT, and had severe deterioration in the lung opacities, with bilateral consolidation described as possible ARDS and/or nosocomial infection. As she showed no signs of clinical improvement despite corticosteroids and High Flow Oxygen Therapy (HFOT), she was intubated, had a repeat bronchofibroscopy and started broad spectrum antibiotics.

The patient developed septic shock and ARDS with refractory hypoxemia and she was put on VV-ECMO. The indirect immunofluorescence was positive for *P. jirovecii* in BAL. She completed 21 days of treatment for PJP and 7 days of piperacillin- tazobactam, with respiratory improvement. ECMO was stopped after 12 days.

Persistent fever and elevated inflammatory markers ensued, with isolation of multidrug-resistant Pseudomonas aeruginosa in respiratory secretions. Chest X-ray confirmed lobar nosocomial pneumonia. She started a targeted antibiotic course with cefepime, with good clinical, analytical, and radiological response. Roughly one week later, she was extubated to non-mechanical ventilation, and rapidly weaned off respiratory support to no oxygen supplementation. The evolution in her condition can be seen at the images in Figure 3b.

She was discharged to the ward after one month of ICU stay for muscular rehabilitation, already on antiretroviral therapy and free of acute infectious complications.

### 2.4. Case 4

A 36-year-old male, overweight and with HIV infection diagnosed in 2009, with poor adherence to appointments and complete discontinuation of ART in the three months before admission.

**Figure 4:**
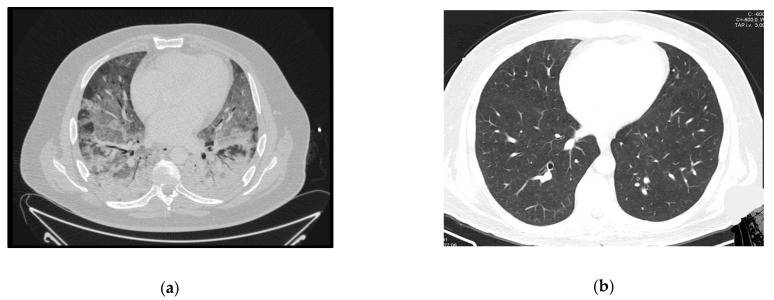
Case 4 thoracic CT-scan at diagnosis (**a**) and follow-up (**b**).

The patient presented at the ER with a 3-week history of worsening cough, dyspnea, and fever. Initial assessment showed hypoxia, fever (39 °C), elevated CRP, 6 CD4+ lymphocytes/mm3 and several ground glass opacities on thoracic CT-scan (Figure 4a). He started empirical treatment with TMP-SMX plus corticosteroids at the recommended PJP treatment dosage and was admitted to the ward. The need for oxygen support increased in the next few hours and the patient responded poorly to HFOT. Twenty-four hours later he was admitted to the ICU and VV-ECMO was started. No tracheal intubation was performed. PJP was confirmed by positive immunofluorescence in BAL.

After 9 days of ECMO support the patient became delirious and agitated, which caused flow problems in the extracorporeal circuit and eventually led to the need for sedation and subsequent intubation. He completed 21 days of treatment, initially with TMP-SMX, then changed to atovaquone plus primaquine due to hematologic toxicity. ECMO support was maintained for 26 days.

He was transferred to the ward for rehabilitation after 37 days of ICU stay, and already on ART. The follow-up CT-scan can be seen in Figure 4b.

All four patients are being followed and regularly observed as part of our Infectious Diseases program and are functional and radiologically recovered, a summary of the patients’ characteristics and evolution is presented in Table 1.

## 3. Discussion

The management of critically ill HIV-positive patients is difficult. However, ICU-related mortality has declined significantly in recent years due to advances in critical support and a better prognosis for HIV patients, associated with advances in ART [3]. A disease that was once thought to be universally fatal in young adults is now a manageable, chronic disease for those responding to therapy. Nevertheless, there is an ongoing debate on the role of the ICU in the management of HIV-positive patients [1].

Late diagnosis is still a problem. In Europe, in 2018, 49% of HIV-infected patients were diagnosed at a late stage, and the same in Portugal, with 34.3% are diagnosed with a T-lymphocyte CD4+ count below 200 cells/mm^3^ and 15.9% are newly diagnosed with AIDS [14].

PJP remains an important risk factor for death in HIV-infected patients and is associated with a lower chance of hospital survival compared to other indications for ICU admission [2]. The need for IMV in HIV-positive patients is also an indicator of poor outcome and often poses a challenge to intensive care physicians making decisions about aggressive ICU support [1]. The use of such resources in this population has often raised ethical and economical questions, and there are still some ICUs that are reluctant to provide organ support to HIV-positive people, a reality that may reflect the impact of outdated studies that persistently report the need for mechanical ventilation in those patients [1].

Importantly, although immunosuppression is an established predictor of mortality for those considered for ECMO, there is little specific data on HIV [5]. With ART, immunosuppression in HIV infection is potentially reversible. The prognosis of patients with HIV-related PJP appears to be better than PJP in patients with other immunosuppressive conditions. [15].

An even more controversial topic is the role of ECMO in improving the outcome of patients with HIV-related ARDS. Although the guidelines of the Extracorporeal Life Support Organization provide no absolute contraindications for ECMO in HIV infected patients, they acknowledge that nonfatal co-morbidities may influence patient selection, whilst other guidelines specifically restrict the role of ECMO in HIV-infected patients [5].

Recently, many papers describing the benefits of ECMO support in HIV-patients with SRF and refractory ARDS have been published [13]. At our center, HIV infection is not an absolute contraindication for ECMO support. However, ECMO is an invasive approach, with an increased risk of contact with the body fluids of patients. This might present a challenge to medical staff caring for these patients.

There is little evidence for ECMO treatment in cases of PJP-induced severe ARDS and information has been reported only in singular case reports and small retrospective studies [13,16,17]. In one retrospective study that included 22 patients with PJP, 6 of them immunosuppressed by HIV, a survival rate of 50% among the HIV-infected patients was reported, which is similar to the average survival of ECMO in patients with ARDS of any origin as shown by the CAESAR (63%) or the EOLIA trial (65%). These data suggest that this therapy should not be withheld from HIV-patients with PJP [17]. There are also other cases in the literature that successfully report the use of ECMO in this particular population [18,19]. In fact, there is growing evidence that the use of ECMO is not necessarily futile in immunocompromised HIV patients. Our case series is consistent with those reports. Furthermore, as ECMO allows more protective ventilation, reducing the risk of barotrauma and VILI, it is conceivable that it might be particularly beneficial for patients with PJP [5].

All of the patients described here had very advanced forms of PJP, with severe ARDS and poor prognosis. In two of our case reports, patients were connected to ECMO prior to intubation and IMV. This strategy of respiratory support was intended to avoid barotraumatic complications, given that these patients with severe lung involvement are prone to pneumatoceles, pneumothorax and pneumomediastinum formation. Sedation sparing would be another potential advantage as ECMO allows for spontaneous breathing with low or no sedation required, thereby leading to less myopathy and delirium. It can also reduce complications of prolonged IMV, such as ventilator-associated pneumonia, VILI, systemic inflammation and multi-organ damage. However, more studies are needed to identify ideal candidates and verify its real advantage [16]. In our non-intubated patients, the tracheal tube was needed later because of ineffective cough and agitation and delirium and this was also described in the cited article.

Of note, all our patients started ART as soon as they tolerated oral intake and after acknowledging their immune status, keeping in mind the possibility of clinical deterioration due to eventual immune reconstitution inflammatory syndrome.

## 4. Conclusions

The clinical course of the presented cases suggests that although immune status is an important factor in the decision to initiate ECMO support, this technology can provide real benefit in some patients with severe HIV-related refractory ARDS. Considering the increasingly reported improvement in survival of patients with HIV infection admitted to the ICU, including those with severe immunosuppression and opportunistic infections, intensive care physicians should change their view and think of including these patients for ECMO support. More studies concerning this specific population are needed. Nevertheless, ICU doctors must always balance the chances of survival with the possible complications and future quality of life of patients submitted to ECMO, regardless of their HIV status.

## Figures and Tables

**Table 1:** Resume of the patients’ characteristics and evolution.

	Case 1	Case 2	Case 3	Case 4
Demographics				
Age (years)	29	64	53	36
Gender	Male	Female	Female	Male
HIV status at admission				
Diagnosis	2015	De novo	De novo	2009
CD4+ T cell count (cells/mm^3^)	6	9	28	6
Viral load (copies/mL)	18.200	4.050.000	673.000	147.000
Previous ART	No	No	No	Yes
Arterial Blood Gas at admission on				
ICU	7.49	7.49	7.44	7.45
pH	49	63	122	73
PaO_2_ (mmHg) PaCO_2_ (mmHg) FiO_2_	32	40	35	36
(%)	30	40	51	85
Ratio PaO_2_/FiO_2_	153	63	239	86
Lactate (mmol/L)	1.3	1.2	4.5	0.7
Number of affected quadrants on Thoracic X-ray	4	4	3	4
Number of days on spontaneous ventilation before intubation	7	0	8	9
Number of days on IMV	60	28	23	18
Prone position	No	Yes	No	No
Need for HFOT (yes/no)	Yes	No	Yes	Yes
Need for Non-Invasive Ventilation (yes/no)	No	No	Yes	No
Number of days on ECMO	41	12	13	26
Length of stay at ICU (days)	68	62	45	37
Mortality scores				
APACHE II	23	35	23	22
SAPS II	25	81	40	49
SAPS III	45	73	48	63
Follow-up 3 months after dischargeCD4+ count(cells/mm^3^) Viral load (copies/mL) Functional status	49 21 Autonomous	43 <20 Autonomous need for domiciliary oxygen therapy (2l/min)	146 23 Autonomous	108 55 Autonomous

Antiretroviral therapy (ART); human immunodeficiency virus (HIV), Intensive Care Unit (ICU), invasive mechanical ventilation (IMV), and high flow oxygen therapy (HFTO).

## Data Availability

No appliable.
